# Little Fingers, Big Problems: Understanding Pediatric Trigger Fingers

**DOI:** 10.7759/cureus.108364

**Published:** 2026-05-06

**Authors:** Olivia C Stanley, Elise Kern, Otto Fisher, Gary Schwartz

**Affiliations:** 1 Orthopedic Surgery, Nova Southeastern University Dr. Kiran C. Patel College of Allopathic Medicine, Fort Lauderdale, USA

**Keywords:** orthopedic hand surgery, pediatric case, pediatric orthopedic surgery, stenosing tenosinovitis, trigger finger release

## Abstract

A trigger finger, also known as stenosing tenosynovitis, occurs when a digit becomes locked in a flexed position due to inflammation of the tendon sheath, restricting smooth tendon excursion. Pediatric trigger fingers (PTF) are less common and consequently less well-characterized - frequently classified as congenital - although the etiology remains heterogeneous and incompletely understood. In this case study, we explore the case of a three-year-old male with flexion deformities of bilateral long and ring fingers. The patient underwent two trigger finger surgeries; the first was a release of the A-1 pulleys, and the second involved a Bruner incision and the excision of the ulnar slip of the flexor digitorum superficialis tendon in all four digits. This case underscores the clinical and surgical complexity of PTF and the corresponding need for more nuanced workup and surgical procedure. Through this exploration, as well as through further investigation of previously published studies, we can appreciate PTF as heterogeneous mechanical disorders rather than a single strict definition.

## Introduction

Pediatric trigger fingers (PTF) are arguably even more elusive than adult trigger fingers. They are less frequently encountered than adult trigger fingers, with trigger thumbs significantly more prevalent than trigger fingers in children [1]. However, PTF are also less easily categorized than adult trigger fingers because, unlike adult trigger fingers, which are generally more inflammatory and occur later in life, PTF are more anatomically heterogeneous and clinically unpredictable. This makes it difficult to believe that PTF are a unified entity [2,3].

The confusion is also evident in the nomenclature of the condition. The term "congenital" is often applied to trigger fingers in children, but it is also evident that many of them fail to demonstrate obvious anatomical abnormalities and instead display a wider range of anatomical variations and mechanical discrepancies in the flexor mechanism [2]. Proximal decussating flexor digitorum superficialis (FDS) tendons, thickened pulley systems, intrinsic tendon changes, and abnormal relationships between the FDS and profundus tendons are some of the anatomical variations that are cited as potential causes of PTF. Therefore, it is evident that trigger fingers are likely to be a complex interplay between tendon and sheath disease rather than a classic obstructive cause of trigger fingers [4].

Although interesting, this proposed complexity has some practical implications. Pediatric trigger thumbs have been observed as more predictable in their response to isolated release of the A1 pulley, but this is not the case with PTF, which have been noted to not follow the same pattern, with some surgical series demonstrating persistence or recurrence after isolated release of the A1 pulley, thus again emphasizing the need to carefully explore the flexor tendon sheath, to carefully identify other mechanical constraints [1,4]. Non-surgical management has been noted to have had even less successful outcomes, with spontaneous resolution being almost negligible, but less so than in trigger thumbs [5].

Thus, the overall message from the literature is that PTF are not so much a distinct clinical entity but a rich spectrum of mechanical disorders in the developing flexor apparatus. In this paper, we present a case of bilateral long- and ring-finger triggering in a young child complicated by the presence of flexion contractures after a previous isolated A1 pulley release, thus again emphasizing the need to carefully evaluate the resolution of PTF.

Evidence from newborn screening studies challenges the label “congenital” for most trigger digits: in a prospective study of 1,046 newborns, Rodgers and Waters [6] identified no trigger digits, estimating an upper bound of zero to three cases per 1,000 live births and noting that surgical presentations occurred after the first months of life, raising the possibility that trigger digits are acquired after birth [6]. Moon et al. [7] similarly examined 7,700 newborns prospectively and also found no congenital cases. This data supports the clinical reality that infants may present within the first year, but true congenital presentations are likely rare and heterogeneous.

Across broader pediatric ages, the most comprehensive etiologic systematic review synthesized 51 studies (193 patients, 398 trigger fingers) and found that 54% presented with a single, unilateral trigger finger while 29% had an underlying condition- most commonly mucopolysaccharidosis/related lysosomal storage disease - often associated with bilateral or multiple trigger digits, carpal tunnel syndrome, or dysmorphic features [8]. Traumatic history was reported in ~5% of the systematic review cohort: these cases tended to be older children with an isolated digit [8]. In the largest multicenter cohort to date (321 patients and 449 PTF), the mean symptom onset was 5.4 years; multidigit involvement, higher Quinnel grade, and palpable nodularity were associated with selection for surgery, suggesting that presentation severity and digit burden influence management pathways [9].

## Case presentation

A three-year-old male presented with flexion deformities of the bilateral long and ring fingers since three months of age. At two years of age, he had surgery in another state, at which time a release of the A1 pulleys of the four digits was performed. There was persistent triggering and the same deformity of the fingers after the initial surgery. Over the next one and a half years, the deformities persisted and were fixed. Being that the patient had minimal active motion, there was no triggering present. There was no history of lysosomal storage disorders, developmental delay, juvenile idiopathic arthritis (JIA), trisomy conditions, or trigger fingers in other children in the family.

**Figure 1:**
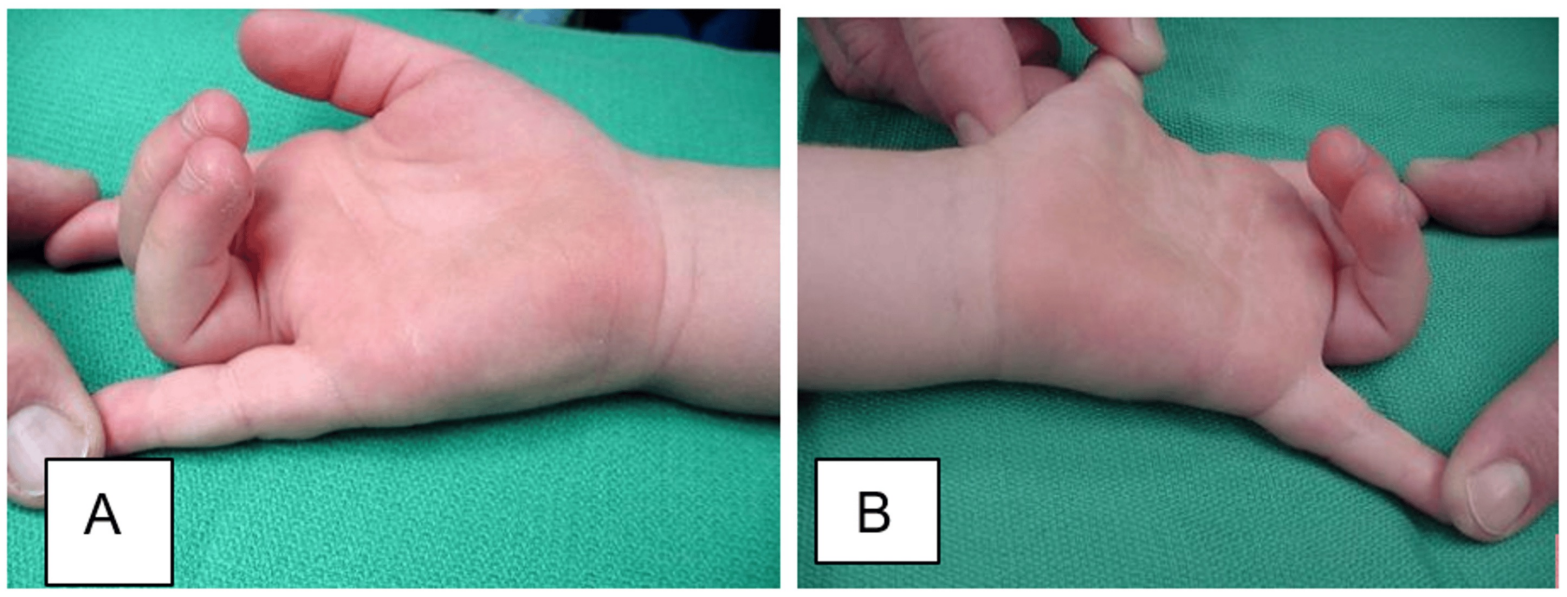
Preoperative photographs of the hands Preoperative photographs of the right (A) and left (B) hands demonstrating the flexion deformities of the long and ring fingers on both hands.

On physical examinations, the patient had flexion deformities of the bilateral long and ring fingers (Figure 1).

The active range of motion of the digits was 0-75 degrees at the metacarpophalangeal joints, 80-115 degrees at the proximal interphalangeal joints, and 0-60 degrees at the distal interphalangeal joints. Passively, the proximal interphalangeal joints could not be extended past 80 degrees of flexion. The proximal interphalangeal motion was not affected by the position of the metacarpophalangeal joints. Radiographs of the hands did not demonstrate any bony or joint pathology.

**Figure 2:**
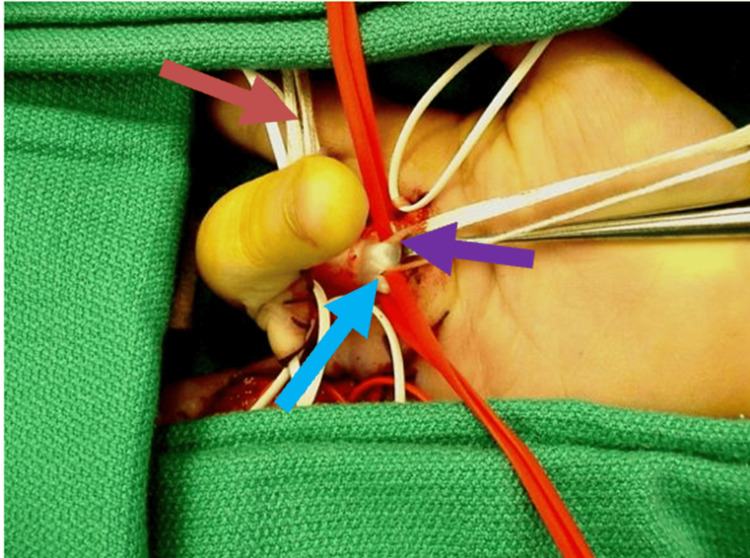
Intraoperative photograph of the right hand An intraoperative photograph of the right hand identifies the ulnar slip of the flexor digitorum superficialis tendon which was excised (blue arrow). The white vessel loupes are identifying and protecting the digital nerves (orange arrow). The flexor digitorum profundus is identified as well (purple arrow)

The patient was taken to the operating room, at which time an extensile Brunner incision was made along the entire flexor tendon sheath of all the affected digits [10]. This approach provided full exposure of the pulley system while allowing safe neurovascular protection. The surgery was performed under general anesthesia in the supine position under tourniquet control. There was scarring in the area of the A1 pulley on all the affected digits. Critically, persistent mechanical obstruction was identified distal to the previously released A1 pulley. There was triggering noted at the A2 and A3 pulleys. No nodules were identified at the A2 pulley location. The ulnar slip of the flexor digitorum superficialis tendon of all the affected digits was excised (Figure 2).

**Figure 3:**
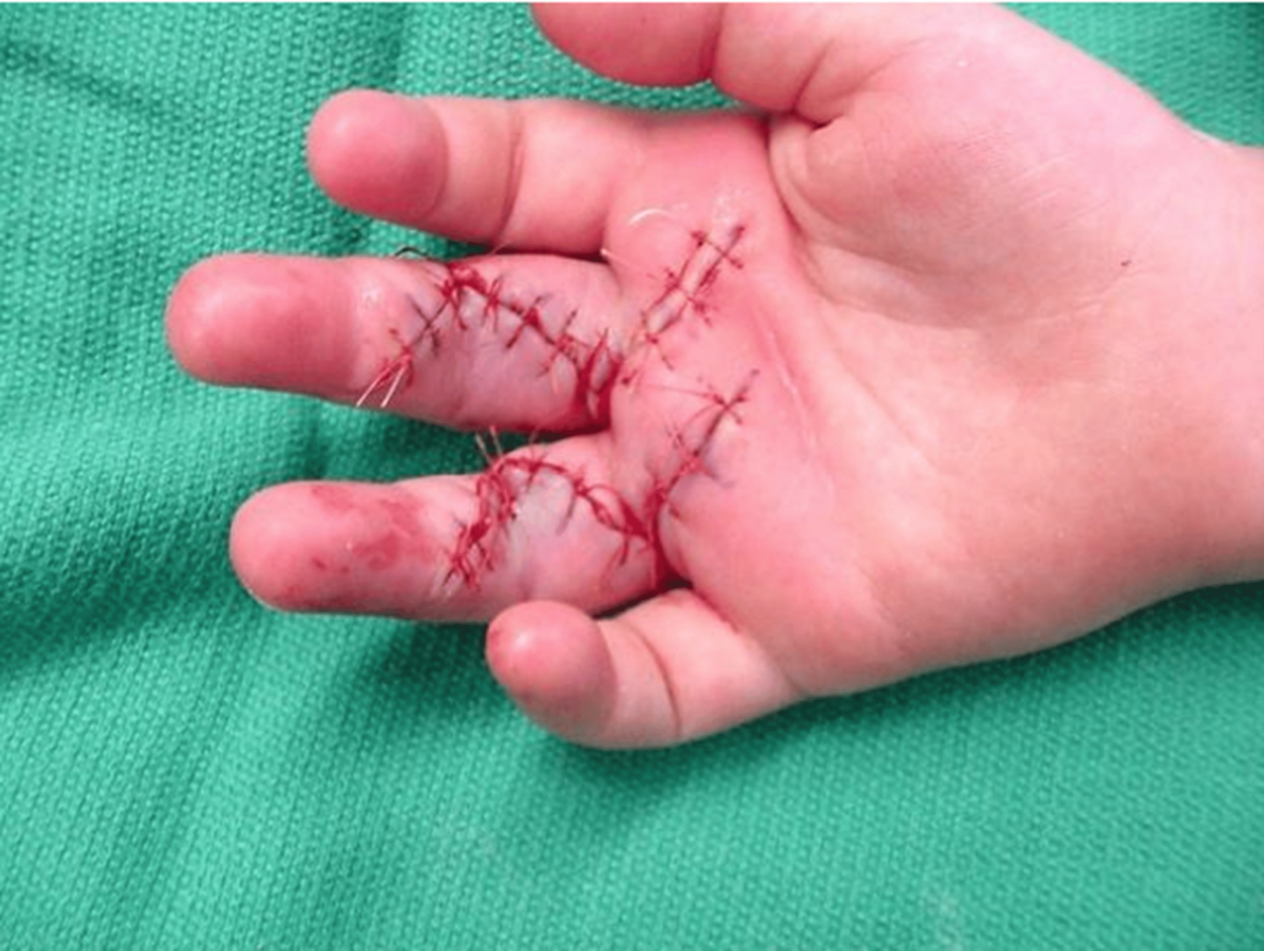
Postoperative photograph of the right hand Postoperative photograph of the right hand demonstrating the extension of the long and ring fingers and the Bruner incision that was utilized for the procedure.

At the end of the procedure, there was no further triggering present, and there was a full passive range of motion of all the digits bilaterally (Figure 3).

A soft dressing was applied, and the patient was allowed to use the hands and digits as tolerated. He was followed up at one week, two weeks, five weeks, and nine weeks, at which time he was discharged from active care. At that time, his range of motion was 0-75 degrees at the metacarpophalangeal joints, 0-110 degrees at the proximal interphalangeal joints, and 0-60 degrees at the distal interphalangeal joints.

Permission was obtained from the patient's family regarding the use of patient images.

## Discussion

PTF are best understood as a clinical phenotype (triggering, locking, or fixed flexion posture of a non-thumb digit) produced by multiple possible mechanical and systemic substrates, rather than a single uniform disease. Contemporary reviews emphasize that the PTF are distinct from the pediatric trigger thumb and adult trigger digits, with less predictable anatomy at the site of obstruction and greater need for individualized diagnostic and surgical planning [11,8]. Recent operative technique guidance likewise frames PTF as uncommon and anatomically complex, recommending structured severity grading (Quinnell scale) to standardize communication and treatment decisions [12].

This case highlights the clinical and surgical complexity of PTF and reinforces the growing understanding that they represent a heterogeneous group of disorders rather than a single, uniform pathology. The significance of this case lies in the failure of isolated A1 pulley release and the subsequent identification of more distal mechanical obstruction involving the A2 and A3 pulleys, as well as the FDS tendon. This directly supports the evolving literature suggesting that PTF often involve multilevel pathology and may not respond adequately to the standard surgical approach used for adult trigger fingers [13,1,14].

Pathophysiology

From a pathophysiologic standpoint, PTF differ fundamentally from adult trigger fingers, which are typically driven by inflammatory thickening of the A1 pulley. By contrast, pediatric cases are more frequently associated with developmental or anatomical abnormalities of the flexor tendon system, including aberrant FDS tendon slips, pulley thickening beyond the A1 level, and altered tendon-gliding mechanics [2,11,4]. In this patient, the persistence of triggering after A1 release and the intraoperative finding of obstruction at the A2 and A3 pulleys suggest that the underlying pathology was not a simple pulley constriction but rather a complex interaction between tendon morphology and sheath mechanics. The excision of the ulnar slip of the FDS tendon, which restored smooth tendon gliding and full passive range of motion, further supports the role of intrinsic tendon abnormalities contributing to mechanical impingement [13,4].

In addition, the development of fixed flexion contractures in this case underscores the consequences of delayed or incomplete resolution of mechanical obstruction. Chronic restriction of tendon excursion likely led to adaptive shortening of surrounding soft tissues and joint structures, resulting in loss of active motion and the absence of classic triggering. This progression emphasizes that PTF may evolve over time from a dynamic triggering condition into a fixed deformity, further complicating both diagnosis and management [9,12].

Underlying mechanisms and how they guide diagnosis

Mechanistically, PTF appear less reliably reducible to “isolated A1 stenosis” than adult trigger fingers, which are typically conceptualized as stenosing tenosynovitis at the A1 pulley and less uniform than pediatric trigger thumb, where developmental mismatch between tendon and pulley is often invoked. Other pediatric surgical series highlight this anatomic diversity: Tordai and Engkvist [14] reported that surgery often required more than A1 release, including separation of FDS slips and release of the proximal A2 pulley, and noted that, unlike trigger thumbs, nodules were not found in their PTF cases [14]. Cardon et al. [1], in addition to describing the distinction between PTF and thumb, also concluded that A1 release alone will not always correct pediatric triggering and that additional steps (e.g., FDS slip resection, A3 release) may be required. [1].

More recent series and operative guideline papers provide convergent evidence that obstruction frequently localizes to the FDS decussation (Camper chiasm) or other anatomic variations: Bae et al. [13] reported that triggering occurred at the level of the FDS decussation in almost half of cases, and they identified associated tendon pathology, including fusiform thickening, nodular thickening, calcific tendonitis, and cyst formation [13].

## Conclusions

A PTF should be understood as a heterogeneous mechanical disorder rather than a single, clearly defined condition. This case of persistent bilateral long and ring finger triggering following isolated A1 pulley release demonstrates that incomplete treatment may result when the pathologic focus extends beyond the A1 pulley. The identification of persistent triggering at the A2 and A3 pulleys, as well as the subsequent successful treatment with excision of the ulnar slip of the flexor digitorum superficialis tendon, underscores the importance of comprehensive exploration in refractory or recurrent cases. Surgeons treating PTF should maintain a high index of suspicion for multifactorial tendon-pulley pathology and tailor operative management accordingly. A more individualized surgical approach may improve functional outcomes and reduce the risk of persistent deformity or recurrence. This case underscores the importance of comprehensive intraoperative evaluation and tailored surgical management in PTF, particularly in the setting of failed prior intervention, where addressing more distal pulley involvement and intrinsic tendon abnormalities may be necessary to achieve durable resolution.
